# Investigation of Serum Neurofilament Light Chain as a Surrogate for Cerebrospinal Fluid Neurofilament Light Chain in Canine Cognitive Dysfunction Syndrome

**DOI:** 10.3390/ani16172659

**Published:** 2026-08-25

**Authors:** Chih-Ching Wu, Wei-Hsiang Huang, Ya-Pei Chang

**Affiliations:** 1 National Taiwan University Veterinary Hospital, College of Bioresources and Agriculture, National Taiwan University, Taipei 106328, Taiwan; chihching@ntu.edu.tw; 2 Institute of Molecular and Comparative Pathobiology, School of Veterinary Medicine, National Taiwan University, Taipei 106319, Taiwan; whhuang@ntu.edu.tw; 3 Institute of Veterinary Clinical Sciences, School of Veterinary Medicine, National Taiwan University, Taipei 106328, Taiwan

**Keywords:** dementia, neurodegeneration, biomarker, senior care

## Abstract

The serum neurofilament light chain (NfL) is a promising circulating biomarker for canine cognitive dysfunction syndrome (CCDS). This cross-sectional study evaluated serum NfL (sNfL) as a surrogate for cerebrospinal fluid NfL (cNfL) and assessed the effects of physiological factors on its clinical utility. Biofluid NfL was analyzed using the single-molecule array (Simoa) across 126 clinical samples, comprising 44 cNfL and 25 paired cNfL/sNfL samples in geriatric dogs (>8 years) and 16 paired samples from a younger cohort (≤8 years). A moderate monotonic correlation was observed between cNfL and sNfL in geriatric dogs, but this association was not maintained in the younger cohort after adjusting for body weight. In the geriatric cohort, raw sNfL levels differentiated cerebral atrophy from controls but not from other intracranial pathologies. Notably, the ability of cNfL to discriminate structural, atrophic, and mixed brain pathologies from controls significantly improved after adjustment for confounding effects of age and body weight. A similar optimization strategy may be applicable to sNfL. Systematically controlling for these physiological covariates in larger cohorts could potentially further improve the diagnostic precision of blood-based NfL screening in primary veterinary practice.

## Simple Summary

Early detection of canine cognitive dysfunction syndrome is often hindered because the gold-standard diagnostic methods, advanced brain imaging and cerebrospinal fluid analysis, require general anesthesia, which poses potential concerns for aging dogs. By analyzing diagnostic samples from clinical veterinary patients, this study evaluated whether a less invasive blood test measuring a protein called neurofilament light chain could act as a reliable surrogate for cerebrospinal fluid tests. We found that while blood and cerebrospinal fluid levels correlated in older dogs, the ability of raw blood and cerebrospinal fluid measurements to discriminate brain pathologies from control dogs was only fair. Notably, we found that the diagnostic power of the cerebrospinal fluid biomarker to identify the presence of brain pathologies was significantly enhanced when physiological variables, such as age and body weight, were statistically adjusted. A larger sample size is required to verify if this optimization strategy is applicable to blood tests, which could ultimately help primary care veterinarians safely identify which vulnerable patient requires advanced imaging.

## 1. Introduction

Canine cognitive dysfunction syndrome (CCDS) is a neurodegenerative disorder similar to Alzheimer’s disease (AD). At the molecular level, CCDS is primarily driven by the accumulation of neurotoxic amyloid-beta (Aβ42) peptides, which are identical in amino acid sequence to those in humans [1,2,3]. In the canine brain, Aβ42 aggregates into diffuse parenchymal plaques and deposits within vessel walls as cerebrovascular amyloid angiopathy [4]. This primary amyloidosis triggers secondary downstream pathological cascades, including microglial and astroglial neuroinflammation, oxidative stress, mitochondrial dysfunction, and progressive synaptic and axonal degeneration [5,6]. However, unlike human AD, the role of Tau protein and neurofibrillary tangles in CCDS remains unclear due to conflicting findings [7]. CCDS is common in dogs aged > 8 years and is characterized by progressive cognitive decline, behavioral changes, and daily functional impairment [2]. CCDS affected 8.1% of dogs aged 8–11 years, 18.8% of dogs aged 11–13 years, 45.3% of dogs aged 13–15 years, 67.3% of dogs aged 15–17 years, and 80% of dogs aged >17 years [8]. Currently, the ante-mortem diagnosis of CCDS is standardized to two levels of certainty. Level 1 certainty is typically established in a clinical setting based on a consistent history of progressive behavioral changes across the DISHAA domains (disorientation, social interactions, sleep distribution, house soiling, learning/memory, and anxiety), along with the exclusion of alternative medical causes through physical and neurological examinations. To achieve level 2 certainty, other intracranial diseases should be ruled out using brain magnetic resonance imaging (MRI) and cerebrospinal fluid (CSF) analysis. Cerebral atrophy, which manifests as a reduction in cortical volume and enlargement of the CSF-filled space, serves as a hallmark of level 2 diagnosis [7]. Specifically, the interthalamic adhesion (IHA) thickness/brain height ratio was the most independent parameter among all age groups when the covariates of sex, body weight (BW), and skull types were considered [9]. However, despite its diagnostic value, the high cost, concerns about anesthesia risk, and benefits to the treatment plan have hindered its application as a routine diagnostic method in primary veterinary practice [2]. Although several fluid biomarkers—including amyloid-beta (Aβ), tau protein, glial fibrillary acidic protein (GFAP), and neurofilament light chain (NfL)—have been explored in CCDS, their clinical diagnostic utility differs markedly. Peripheral measurements of Aβ42, Aβ40, and their ratios across aging and CCDS cohorts have produced conflicting results [10,11,12,13,14]; recent meta-analyses demonstrate that circulating concentrations lack sufficient consistency to serve as a standalone diagnostic marker [15]. Similarly, blood levels of tau protein [16], and GFAP [13,14] show significant overlap among normal aging and cognitively impaired dogs, demonstrating no reliable correlation with owner-reported questionnaires or clinical diagnoses [16]. In contrast, NfL levels in blood consistently display strong, reproducible correlations with advancing age, behavioral questionnaire scores, and neurodegenerative disease severity [16,17,18,19].

Neurofilaments are major structural elements of neurons that are present in the perikarya and dendrites and are particularly abundant in large myelinated axons [20]. NfL is a subunit of neurofilaments and has evolved into the most promising biomarker to indicate neuro-axonal damage, being at the doorstep of clinical application in the last two decades [21]. Under normal conditions, low levels of NfL are constantly released from axons into biofluids and increase in a strong, non-linear relationship with age [22]. In response to axonal damage, NfL is released from neurons into the interstitial fluid and enters the free-communicating CSF compartment [23]. It is believed that the primary source of blood NfL is CSF passing through a damaged blood–brain barrier [24]. Clinical findings from human studies have shown that its levels increase in CSF and blood in proportion to the degree of axonal damage in a variety of neurological disorders, including inflammatory, neurodegenerative, traumatic, and cerebrovascular diseases [23]. However, previous studies have found that CSF NfL is more sensitive in the early diagnosis of preclinical sporadic AD [25] and in tracking the dynamic progression of neuronal injury in the later symptomatic stages of AD [26]. In dogs, NfL concentration in the CSF and/or blood has been evaluated in various diseases, including CCDS [17,18], meningoencephalitis of unknown etiology (MUE) [27,28], hydrocephalus [29], idiopathic epilepsy [28], brain tumors [28,29], neuronal ceroid lipofuscinosis type 2 [30], Chiari-like malformation/syringomyelia [28], myelitis [28], and degenerative myelopathy [18]. Its value is recognized in distinguishing structural brain diseases from idiopathic epilepsy [29], predicting lesion sizes of MUE and hydrocephalus [27], monitoring treatment responses in neuronal ceroid lipofuscinosis types 2 [30] and MUE [27], and diagnosing CCDS [17,18].

Though the use of blood-based NfL concentration in the diagnosis of CCDS shows promise and, when considering the risks of anesthesia, has obvious advantages over CSF-based NfL measurements and neuroimaging, it should be noted that NfL is a sensitive but nonspecific marker of axonal damage [31,32], and its concentration is susceptible to various confounding factors during its dilution, degradation, and clearance in blood [21,33]. In dogs, its ability to discriminate MUE from hydrocephalus and brain tumors was evident in one study [29] but has not been evaluated in neurodegenerative versus non-neurodegenerative diseases. In addition, the use of blood NfL as a surrogate for CSF NfL in the CCDS requires further investigation. The overall pooled correlation coefficient estimate between CSF and blood NfL levels across pathologies was moderately strong. However, blood NfL levels do not correlate perfectly with CSF NfL levels, and additional factors may need to be considered when interpreting blood NfL results [24]. Strong associations between cross-sectional CSF and blood NfL exist in patients with autosomal dominant AD, but the correlation between sporadic AD and healthy older adult populations is heterogeneous, suggesting that different pathological processes contribute partly to the dispersion [26]. Blood NfL concentrations become more variable in humans older than 60 years of age [22]. In this population, renal function has been identified as the second most important factor affecting blood NfL concentrations after age, whereas no such association has been observed in the younger age group [21]. In dogs, the association of the covariates and blood NfL levels, including age, sex, neuter status, BW, height, body mass index, and coat color, has been evaluated [18,19,34]. Previous studies have established that sex and neuter status do not significantly influence blood NfL concentrations [18,19]. Age was consistently the most important covariate of blood NfL level [18,19,34]. In addition to age, only BW and height were found to have a negative association in healthy Labrador retrievers younger than 11 years of age, but this association varied in the older group [19]. In another study, no correlation between BW and blood NfL was observed, and height was not appraised [18]. It remains unclear whether these factors affect NfL levels differently among life stages, and whether the correlation between CSF and blood NfL in aged dogs remains consistent. This study aimed to assess the feasibility of utilizing blood NfL concentration as a practical surrogate biomarker for CSF NfL in the diagnosis of CCDS in clinical settings, while systematically examining the influence of potential covariates on CSF and blood NfL levels and their interrelationship. We hypothesized that (1) significant differences in CSF and blood NfL concentrations exist among geriatric dogs with advanced imaging-confirmed cerebral atrophy, those with other intracranial pathologies, and controls with morphologically normal brains on MRI; (2) the correlation between CSF and blood NfL levels varies across different age cohorts; and (3) the correlation between CSF and blood NfL levels is influenced by physiological factors, including age, renal function, and BW.

## 2. Materials and Methods

### 2.1. Blood NfL Samples

Previous canine studies on blood NfL have predominantly used plasma samples. However, serum and plasma NfL concentrations are strongly correlated [35], and both matrices have been widely studied in human studies [36]. Given that serum samples are routinely collected for pathogen screening in dogs undergoing MRI and CSF examinations at our institution, serum NfL concentration was selected as the biomarker for this study.

### 2.2. Case Selection

This was a single-center, retrospective, cross-sectional study. All dog owners provided informed consent for the use of their dogs’ clinical data. Medical records from 2007 to 2024 were reviewed to identify dogs > 8 years of age that had undergone MRI and CSF analysis and were diagnosed with CCDS or other brain diseases. This cutoff was selected in accordance with the established veterinary literature evaluating CCDS and biomarkers in demographically diverse, multi-breed companion dog populations [11,37]. The imaging inclusion criteria were as follows:

1. Brain MRI included the olfactory lobes through at least the level of the second cervical vertebra.
2. The imaging protocol included, at minimum, transverse T1-weighted (T1W), T2-weighted (T2W), fluid attenuated inversion recovery, and post-contrast T1W sequences, as well as sagittal T2W and post-contrast T1W sequences.
3. Transverse T1W and T2W images at the level of the IHA were clear to allow measurement of the IHA thickness-to-brain height ratio.

Following MRI characterization and confirmation of inclusion criteria, the institutional biobank repository was searched to retrieve matching residual cerebrospinal fluid (CSF) or paired residual CSF and serum samples. Selected CSF samples spanned collection dates from April 2014 to October 2024, whereas paired serum samples were collected between January 2021 and October 2024 and maintained continuously prior to execution of single-molecule array (Simoa HD-1 analyzer, Quanterix Corporation, Billerica, MA, USA) testing within 3 months of collection. Although a positive association between CSF and blood NfL concentrations has been reported in young and adult dogs, this relationship in aged dogs may have different dynamics. To provide a comprehensive assessment, a comparison group consisting of 16 dogs ≤ 8 years of age with brain diseases was included in parallel to assess the inherent covariates of NfL concentrations in body fluids that may be masked after aging. These dogs met the same imaging criteria and had paired residual CSF and serum samples available.

To evaluate physiological factors influencing NfL concentrations in dogs entering the risk window for CCDS, study participants were stratified into two main age cohorts: a younger cohort (≤8 years; *n* = 16) and a geriatric cohort (>8 years; *n* = 69). All clinical diagnoses and neuroimaging classifications were established independently of and prior to biofluid biomarker analysis. In the younger cohort, 16 paired cNfL and sNfL samples are collected. In the geriatric cohort, 25 provided paired CSF and serum samples, while 44 provided only CSF samples.

### 2.3. Imaging Review

The MRI studies were independently reviewed by a board-certified neurologist and a neurology resident. The IHA thickness/brain height was calculated according to a previous study, and a ratio of <13.6% was considered as cerebral atrophy [9]. Four imaging-based subgroups were established based on the imaging findings as follows:

1. Structural brain diseases (SBD): dogs with extra- and/or intra-axial lesions and an IHA thickness/brain height ratio greater than 13.6% were included.
2. Cerebral atrophy (CA): dogs with an IHA thickness/brain height ratio less than 13.6% and no other extra- or intra-axial lesions were included in this subgroup.
3. Mixed: dogs with extra- and/or intra-axial lesions and an IHA thickness/brain height ratio less than 13.6% were included in this subgroup.
4. Control: dogs with morphologically normal brains (absence of abnormalities in morphology and signal intensity in all provided sequences), an IHA thickness/brain height ratio greater than 13.6%, and normal CSF analysis (unremarkable cytology and protein) were included in this subgroup.

### 2.4. The Assessment of the Covariates

Data, including age, body weight, and plasma creatinine levels, were extracted from medical records. Based on International Renal Interest Society (IRIS) guidelines, CKD staging of chronic kidney disease was designated for dogs based on their plasma creatinine levels.

### 2.5. Sample Analysis

Blood and CSF samples were collected in Eppendorf tubes. The blood was then centrifuged. An amount of 500 µL of CSF and serum were aliquoted and sent for the routine CSF analysis and/or pathogen screening tests. The residues of the CSF and serum were stored at −80 °C for future analyses.

CSF (cNfL) and serum NfL (sNfL) concentrations were measured using the NF-Light Advantage PLUS assay with a single-molecule array (Simoa) on an HD-1 analyzer, according to the manufacturer’s instructions. The quality control metric of Advantage PLUS includes the lower limit of quantification, which is determined by analyzing serial dilutions of calibrators to confirm acceptable accuracy (80–120% of the expected value) and precision (coefficient of variation < 20%) across multiple runs, reagent lots, and instruments. The detection limit was 0.062 pg/mL. The validated measurement range was 0–1800 pg/mL for serum and plasma and 0–45,000 pg/mL for CSF. Any specimen with an initial concentration exceeding the upper limit of detection was diluted by a specific factor (e.g., 1:10 or 1:100) and re-measured. The final NfL concentration of these samples was calculated by multiplying the dilution result by the corresponding dilution factor.

### 2.6. Statistical Analysis

Data normality for all continuous variables, including cNfL and sNfL concentrations, across the four imaging-based subgroups and both age cohorts (>8 years and ≤8 years) was assessed using the Shapiro–Wilk test. To evaluate the continuous, non-linear influence of age on biofluid NfL levels across the paired dataset (*n* = 41), a Generalized Additive Model (GAM) was constructed using a Gamma distribution with a log-link function. Continuous age and cNfL were fitted using non-parametric thin-plate regression splines (s(Age) and s(cNfL)). Model parsimony, effective degrees of freedom (edf), and partial effect curves were evaluated to test for statistical non-linearity. Non-linear trajectories and potential inflection points across continuous predictors were further evaluated by inspecting scatterplots overlaid with locally weighted smoothing (LOESS) regression lines. The linearity assumption of cNfL and sNfL in both age cohorts was further evaluated by inspecting the scatterplots of continuous predictors against the dependent variable and model residuals, utilizing LOESS to aid interpretation.

Because of the non-normal distribution and non-linearity observed in the dataset, non-parametric statistical methods were employed. The Kruskal–Wallis test followed by post hoc analysis using Dunn’s test with Bonferroni correction was used to compare the cNfL and sNfL concentrations in four imaging-based subgroups (SBD, CA, Mixed, and control) of dogs aged >8 years. The correlation between the cNfL and sNfL levels in both age groups was initially investigated. If an obvious discrepancy existed, the statistical analysis of NfL was performed separately on dogs in different age cohorts, while exploring the correlation between cNfL and sNfL levels and the associated physiological factors. Spearman’s correlation analysis was performed to determine the association between cNfL and sNfL levels, age, BW, and IRIS stage. The relationship between the cNfL and sNfL levels was adjusted for statistically significant factors using a partial Spearman correlation analysis. Finally, a generalized linear model (GLM) specified with a Gamma distribution family and log-link function was computed to test the confounding effects of significant physiological factors and imaging-based subgroups. In these GLM regressions, biofluid NfL concentration served as the dependent outcome variable, imaging-based diagnostic subgroup served as the primary categorical fixed factor, and continuous age and body weight were incorporated as continuous multivariable covariates. Because LOESS and GAM fittings revealed slope heterogeneity across life stages (with biofluid NfL remaining flat at younger ages and accelerating past senior age), GLMs were constructed within age-stratified cohorts (≤8 years vs. >8 years) to adjust for continuous age and body weight within homogeneous biological life stages. No a priori or post hoc sample size calculation or statistical power analysis was performed for this study. The retrospective biobank availability for paired geriatric serum samples (*n* = 25) provided limited post hoc statistical power for unadjusted subgroup comparisons, which is acknowledged as a study limitation in Limitations.

Descriptive statistical analyses were performed for all demographic and clinical variables. Data normality for continuous variables was evaluated using the Shapiro–Wilk test. Because continuous variables (including age, body weight, cNfL, and sNfL) deviated significantly from a normal distribution, continuous data are reported as medians and interquartile ranges (IQR). Categorical variables are reported as absolute frequencies. Where applicable—including partial correlation coefficients, odds ratios, and GLM parameter estimates—two-sided 95% confidence intervals (95% CI) were calculated. Statistical analyses were conducted using commercially available software (SPSS statistical program, IBM SPSS Statistics 25, IBM Corporation, Armonk, NY, USA) and R (version 4.5.1), and graphs were generated using GraphPad Prism 7.00 for Mac (GraphPad Software, La Jolla, CA, USA) and R (version 4.5.1). A *p*-value < 0.05 was considered statistically significant.

## 3. Results

A total of 126 samples were collected for NfL analysis. Among dogs older than 8 years, 25 provided paired CSF and serum samples, while 44 provided only CSF samples. Additional paired samples were collected from 16 dogs aged 8 years or younger.

The median age of the older cohort was 12 years old (IQR: 11–14), and the median BW was 10.4 kg (IQR: 6.2–17.4). There were 23 spayed females, two intact females, 32 neutered males, and 12 intact male dogs. Sixteen breeds were represented in this group as Miniature Poodle (*n* = 15), mixed breed (*n* = 14), French Bulldog (*n* = 7), Shiba Inu (*n* = 7), Miniature Schnauzer (*n* = 5), Welsh Corgi (*n* = 2), Golden Retriever (*n* = 4), Border Collie (*n* = 3), Miniature Dachshund (*n* = 3), Spitz (*n* = 2), Maltese (*n* = 2), and one in each of the following breeds: Labrador Retriever, Bichon Frise, Shih Tzu, Shetland Sheepdog, and Chihuahua. Descriptive statistics and clinical diagnoses of each subgroup are listed in Table 1 and Table 2. In the SBD subgroup, neoplastic diseases represented 23/25 (92%) of the clinical diagnoses in the cNfL analysis and 7/9 (78%) in the sNfL analysis. In the CA subgroup, all dogs were clinically diagnosed with CCDS, representing 18/18 cases in the cNfL analysis cohort and 5/5 cases in the sNfL analysis cohort.

The median age of the younger cohort was 6 years (IQR: 3–6), and the median BW was 11.5 kg (IQR: 6.3–20.2). The cohort comprised four spayed females, two intact females, nine neutered males, and one intact male. Twelve breeds were represented: Bichon Frise (*n* = 3), Border Collie (*n* = 2), Welsh Corgi (*n* = 2), and one each of mixed breed, French Bulldog, Spitz, Shiba Inu, Labrador Retriever, Siberian Husky, Pomeranian, Alaskan Malamute, and Pug. Based on imaging findings, 12 dogs were assigned to the control subgroup and 4 to the SBD subgroup. Idiopathic epilepsy was the predominant clinical diagnosis in the control subgroup (9/12), with the remaining dogs diagnosed with otitis media/interna, diabetes insipidus, or paroxysmal dyskinesia (one each). In the SBD subgroup, three dogs were diagnosed with MUE and one with a cerebellar ependymal cyst.

### 3.1. CSF and Serum NfL Levels in Imaging-Based Subgroups of Dogs > 8 Years Old

**Figure 1:**
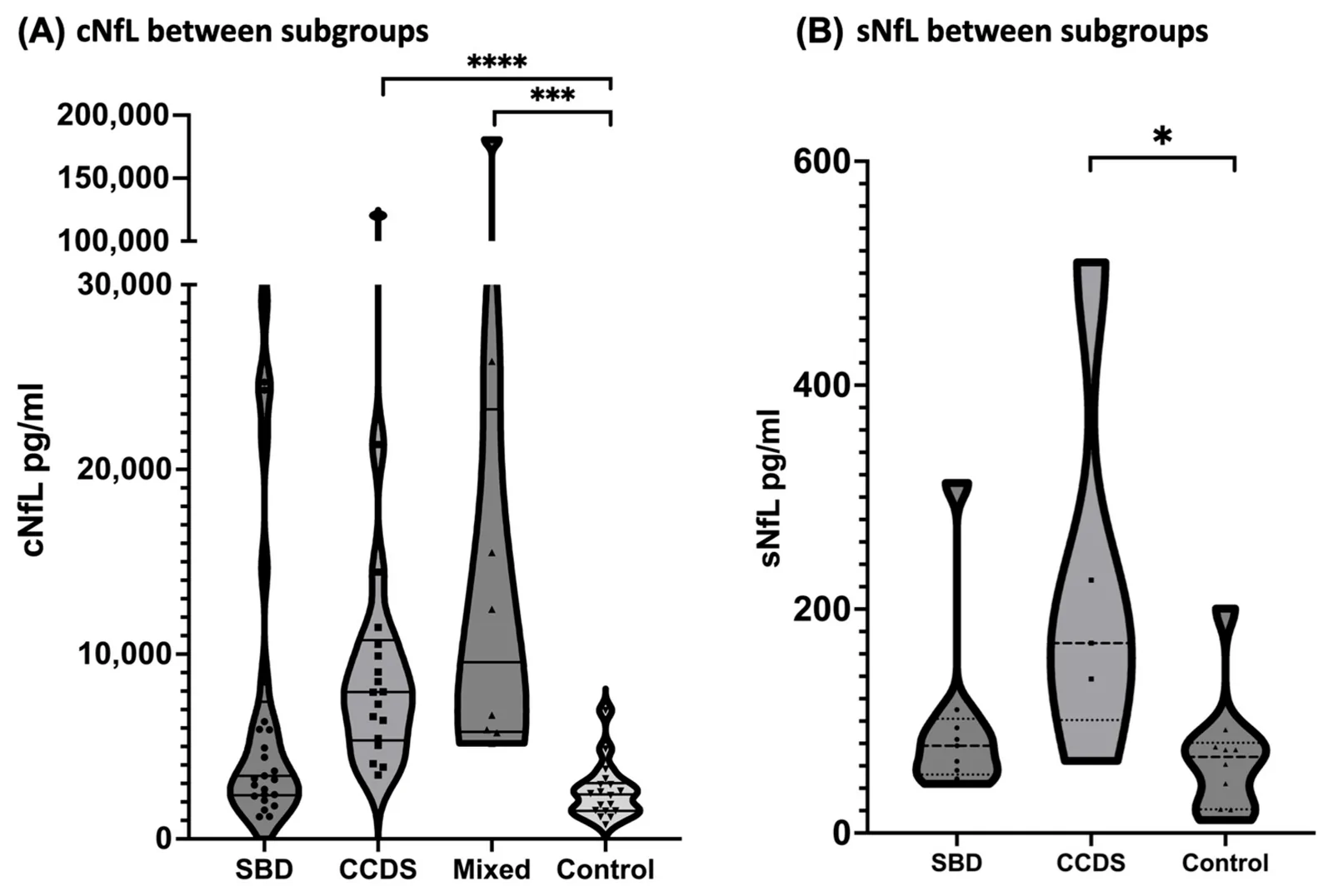
Violin plots with overlaid individual data points comparing neurofilament light chain (NfL) concentrations in cerebrospinal fluid (**A**) and serum (**B**) across imaging-based subgroups of dogs > 8 years of age. (**A**) Cerebrospinal fluid NfL (cNfL) levels differed significantly between the CA and control subgroups (*p* < 0.0001) and between the mixed and control subgroups (*p* < 0.001). (**B**) Serum NfL (sNfL) levels differed significantly between the CA and control subgroups (*p* = 0.034). The mixed subgroup was excluded from serum analysis due to small sample size (*n* = 1). Violin outlines depict kernel probability density distributions, internal horizontal lines indicate the median and interquartile range (25th and 75th percentiles), and individual data points represent individual dog observations. CA, cerebral atrophy; cNfL, neurofilament light chain in cerebrospinal fluid; SBD, structural brain disease; sNfL, neurofilament light chain in serum. *, *p* < 0.05; ***, *p* < 0.001; ****, *p* < 0.0001.

A total of 69 cNfL and 25 sNfL samples were analyzed. Pairwise comparisons of cNfL levels showed significant differences between the control and CA subgroups (*p* < 0.001) and between the control and mixed subgroups (*p* < 0.001) but not between the other subgroups (Figure 1A). The difference between the SBD and CA subgroups was borderline significant (*p* = 0.055). Because only one serum sample was classified as the mixed diagnosis, the mixed subgroup was excluded from the sNfL analysis. Serum NfL levels differed significantly between the CA and control subgroups (*p* = 0.034). However, no significant differences were found between the other subgroups (Figure 1B).

### 3.2. Correlation Between the cNfL, sNfL Levels, and Clinical Factors in Paired Dataset, >8 y/o and ≤8 y/o Dog Groups

**Figure 2:**
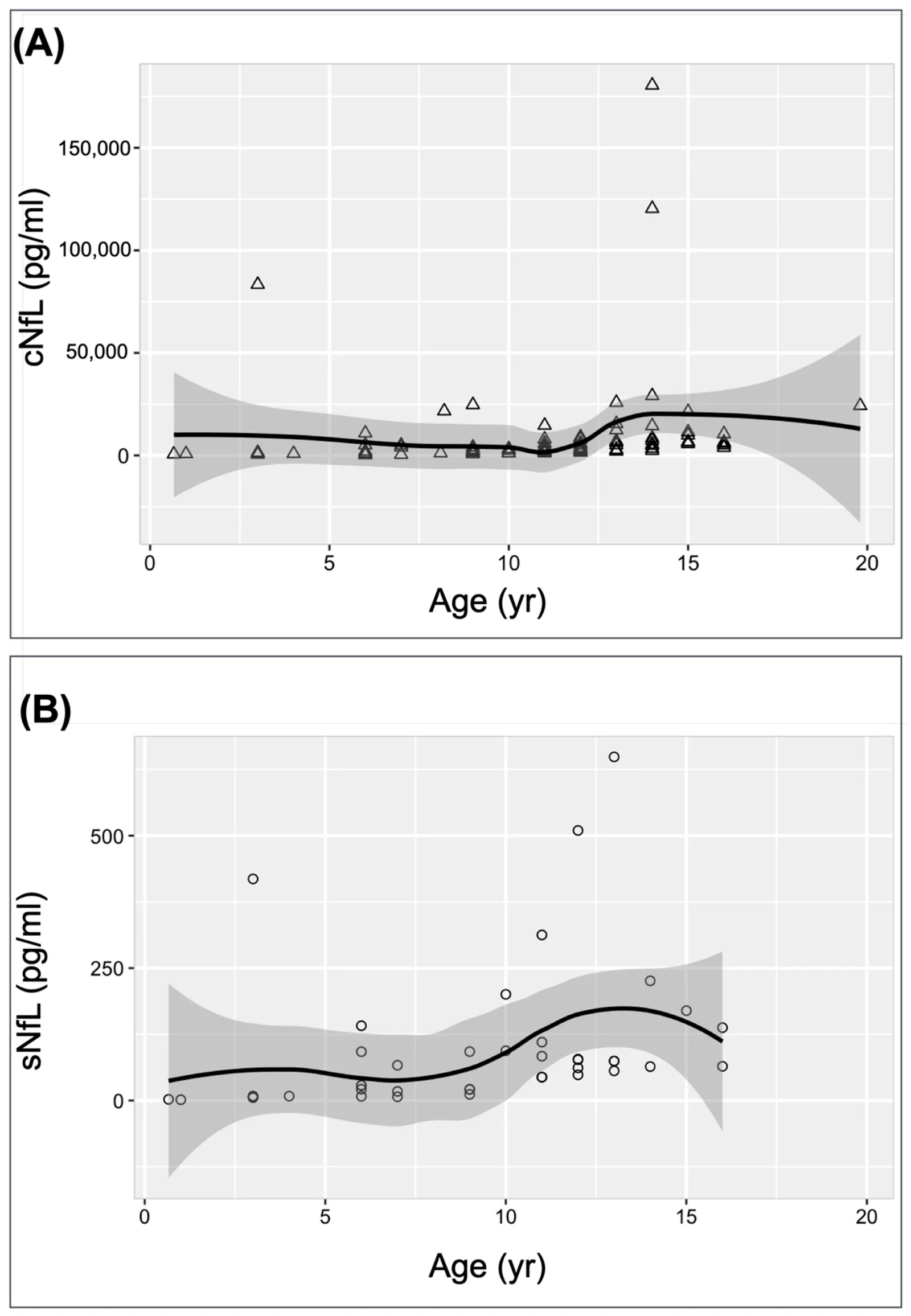
Relationship between continuous age and neurofilament light chain concentrations in cerebrospinal fluid ((**A**), cNfL) and serum ((**B**), sNfL) fitted with non-parametric locally weighted smoothing (LOESS) regression curves. Both central (cNfL) and peripheral (sNfL) concentrations display non-linear, age-dependent accumulation dynamics across the continuous canine lifespan. The trajectories show flat baseline concentrations in younger dogs followed by a distinct upward biological inflection point occurring around 10 years of age, reflecting accelerated subclinical neuro-axonal attrition in senior dogs entering the window of risk for canine cognitive dysfunction syndrome (CCDS). Open triangles in (**A**) and open circles in (**B**) represent individual paired observation data points. CCDS, canine cognitive dysfunction syndrome; cNfL, neurofilament light chain in cerebrospinal fluid; sNfL, neurofilament light chain in serum.

Evaluating continuous age across the unstratified, paired dataset (*n* = 41) using a multivariable Generalized Additive Model (GAM) confirmed that age is a major non-linear driver of biofluid NfL concentrations, consistent with the prior canine neuro-aging literature. The overall GAM accounted for 69.9% of total deviance in sNfL (R^2^ adj = 0.656, GCV = 0.646). Smooth spline terms demonstrated that continuous age exerted a highly significant, non-linear effect on sNfL levels (edf = 2.505, *F* = 11.03, *p* < 0.001), while cNfL remained an independent non-linear predictor (edf = 1.881, *F* = 10.94, *p* < 0.001). Non-parametric LOESS curves on raw concentration scales corroborated these findings, displaying a flat baseline trajectory in younger animals followed by a distinct upward biological inflection point occurring at 11 years of age (Figure 2).

**Figure 3:**
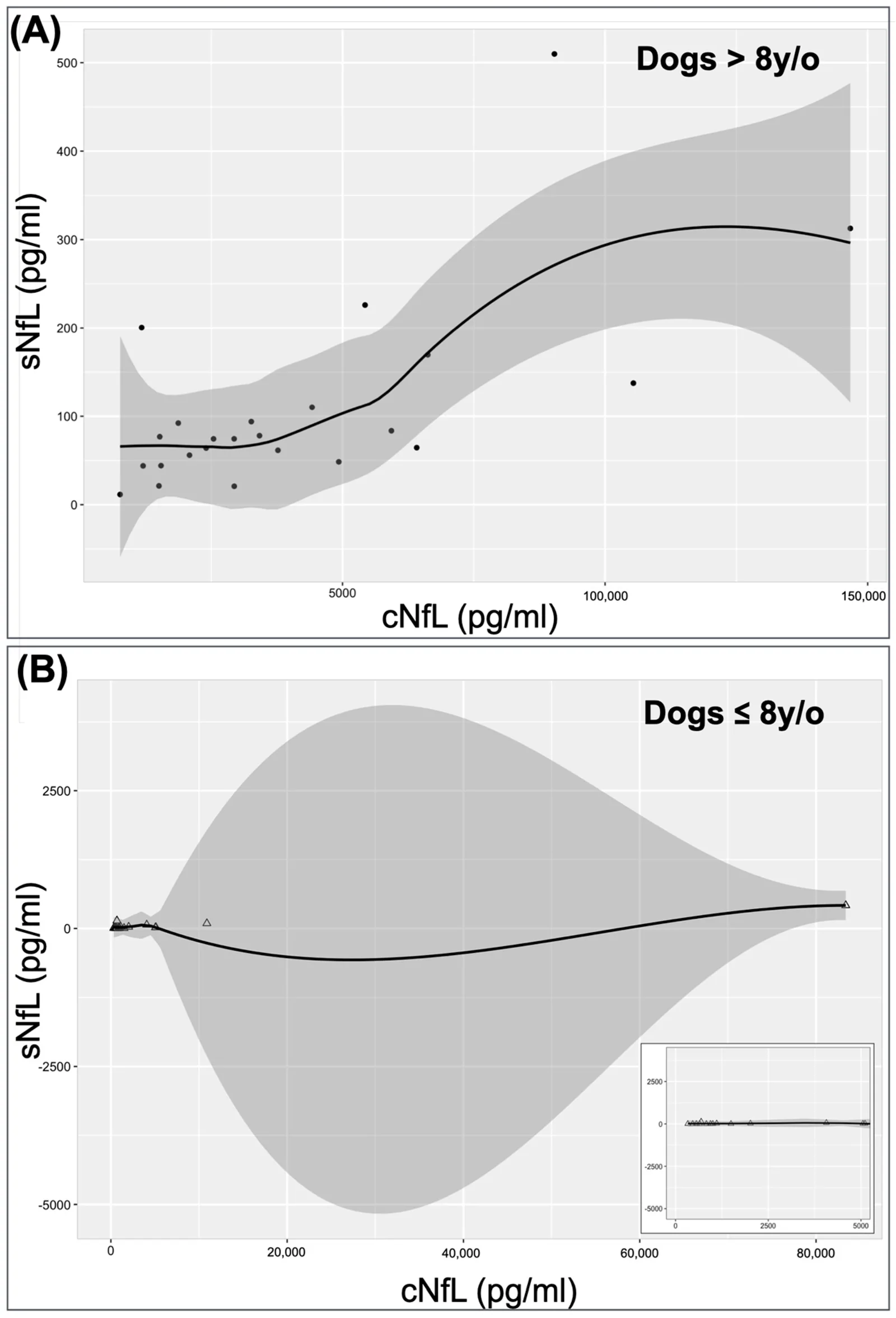
Association between cerebrospinal fluid neurofilament light chain (cNfL) and serum neurofilament light chain (sNfL) in dogs > 8 and ≤8 years of age. Scatter plots with locally weighted smoothing (LOESS) curves illustrate the relationship between cNfL and sNfL in the two age groups. (**A**) Dogs > 8 years show a monotonic correlation. (**B**) Dogs ≤ 8 years show no clear linear correlation. Due to clustering of data points near zero, the LOESS fit line dips slightly below zero. Inset: Zoomed-in view of the primary data cluster in the lower concentration range. Note: The minor dip of the LOESS curve below zero near the origin represents a non-parametric polynomial smoothing boundary artifact resulting from sparse data clustering at low baseline concentrations, rather than a negative biological value or negative association. Distinct correlation patterns are observed between age groups. cNfL, neurofilament light chain in cerebrospinal fluid; sNfL, neurofilament light chain in serum.

The associations between cNfL and sNfL levels in the two age groups are shown in Figure 3. While a clear monotonic correlation between cNfL and sNfL concentrations was observed in the geriatric cohort (>8 y/o; Spearman’s *ρ* = 0.63, *p* < 0.001, 95% CI 0.30–0.83), this relationship was not observed in the younger group (≤8 y/o; Spearman’s *ρ* = 0.57, *p* = 0.021, 95% CI 0.089–0.84). The distinct patterns between the two scatter plots supported the decision to perform separate statistical analyses for different age cohorts.

**Figure 4:**
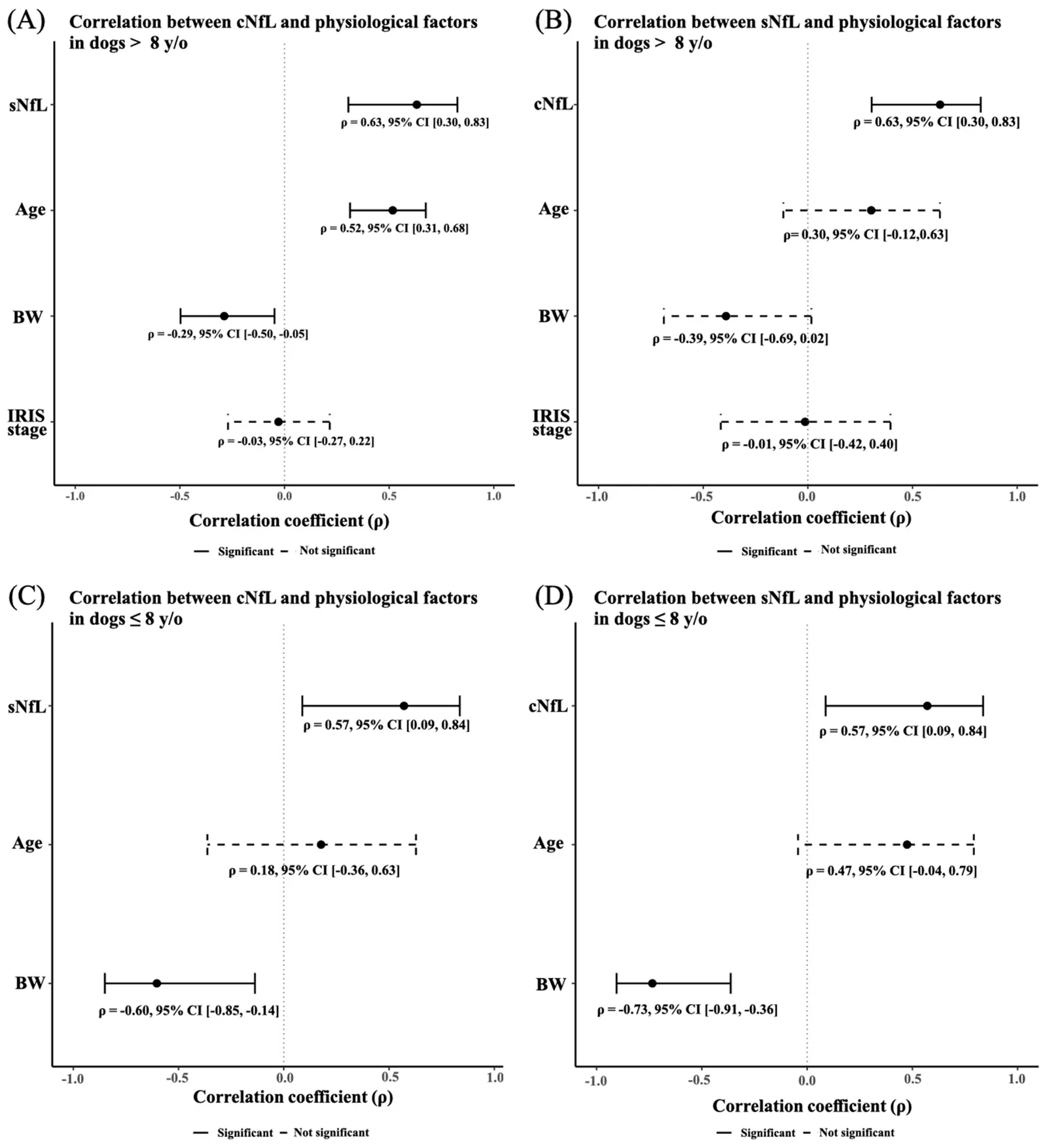
Spearman’s correlation between cNfL, sNfL, and physiological factors in dogs > 8 years (**A**,**B**) and ≤8 years (**C**,**D**) of age. (**A**) In dogs > 8 years of age, cNfL levels were significantly correlated with sNfL (*ρ* = 0.63, *p* < 0.001), age (*ρ* = 0.52, *p* < 0.001), and body weight (*ρ* = −0.29, *p* = 0.016). (**B**) In dogs > 8 years of age, sNfL levels were significantly correlated with cNfL (*ρ* = 0.63, *p* < 0.001). (**C**) In dogs ≤ 8 years of age, cNfL levels were significantly correlated with sNfL (*ρ* = 0.57, *p* = 0.021) and body weight (*ρ* = −0.60, *p* = 0.013). (**D**) In dogs ≤ 8 years of age, sNfL levels were significantly correlated with cNfL (*ρ* = 0.57, *p* = 0.021) and body weight (*ρ* = −0.73, *p* = 0.001). BW, body weight; CI, confidence interval; cNfL, neurofilament light chain in cerebrospinal fluid; IRIS, International Renal Interest Society; sNfL, neurofilament light chain in serum.

Associations between cNfL and sNfL levels and physiological factors, including age, BW, and IRIS stage, were assessed (Figure 4). Due to the lack of variability within the younger cohort, in which all individuals were classified as IRIS stage 1, statistical analysis of IRIS stage in this group was omitted. In the >8 y/o group, age and BW were significantly associated with cNfL levels, whereas IRIS stage was not. In the ≤8 y/o group, BW was significantly correlated with both cNfL and sNfL levels.

Correlations between cNfL and sNfL concentrations were adjusted using partial Spearman analysis. After controlling the effects of age and BW, the correlation between cNfL and sNfL concentrations in the >8 y/o group remained significant but reduced (Spearman’s *ρ* = 0.458, *p* = 0.021). However, the result in the ≤8 y/o group did not remain significant after adjusting for the effect of BW (Spearman’s *ρ* = 0.235, *p* = 0.380), suggesting the strong influence of BW.

### 3.3. Influence of Covariates on NfL Concentrations Across Imaging-Based Subgroups

Based on the correlation test, a generalized linear model (GLM) was used to evaluate the influence of age and BW on cNfL levels in the different subgroups of the older cohort. After adjusting for covariates, the pairwise comparison showed a significant difference between the SBD and control, the CA and control, and the mixed and control subgroups (*p* < 0.001). There were no significant effects of age (*p* = 0.099) or BW (*p* = 0.0839) on cNfL levels after adjusting for the overall model effect of the subgroups. The GLM of the younger cohort indicated that both imaging-based subgrouping and BW influenced the cNfL and sNfL levels. The subgrouping of cNfL reached *p* < 0.001 and sNfL reached *p* = 0.039. The effect of BW on both cNfL and sNfL concentrations remained a dominant factor (both *p* < 0.01) even after controlling for imaging-based subgrouping in the GLM.

## 4. Discussion

In the present study, we found that cNfL was a more robust biomarker than sNfL for intracranial pathologies in dogs aged > 8 years. The correlation between cNfL and sNfL levels was moderate and influenced by factors including age and BW. After adjusting for age and BW, the ability of cNfL to discriminate intracranial morphological changes from normal brain tissue was enhanced.

In the last two decades, growing evidence has been generated regarding the use of blood NfL as a diagnostic biomarker for various CNS disorders in both human and veterinary medicine. Neurofilament light chain is a primary structural component that is particularly abundant within the large-caliber, myelinated axons of long-range neocortical pyramidal neurons [20,38]. When these cognitive-relevant neurons undergo age-associated neurodegeneration, structural decay, and axonal loss, NfL intermediate filaments disassemble and are passively liberated into extracellular biofluids [23,39]. Recent studies in CCDS established blood NfL as a robust translational biomarker [14,16,17,18]. While studies on human neurodegenerative diseases commonly include neuroimaging to support diagnosis and validate blood NfL as a surrogate for cNfL, concerns regarding risks and limited clinical benefits preclude examinations requiring anesthesia in geriatric dogs. Recent studies have identified a discrepancy in CSF and blood NfL levels in sporadic AD and normally aging people [26] and confirmed that blood NfL is influenced by factors including age, renal function, blood volume, and high-density lipoprotein levels at different life stages [13]. This raises the need to validate this biomarker in CCDS by corroborating information from neuroimaging and CSF analyses before clinical application.

Our study found that cNfL concentrations in dogs with structural brain disease and cerebral atrophy were significantly different from those in dogs without morphological changes in the brain. A recognized feature of clinical geriatric canine cohorts is the inherent baseline age disparity across neurological subcategories. In our study, dogs in the CA subgroup exhibited a significantly higher median age (14–15 years) compared to the control (10.5–11 years) and SBD (10.5–11.0 years) subgroups. Because NfL concentrations naturally increase with physiological aging due to subclinical axonal attrition, advanced age acts as an important confounding variable. Although our GLM statistical framework successfully isolated the independent main effect of disease group after controlling for age and body weight (*p* < 0.001), mathematically adjusting for age cannot completely uncouple the biological interplay between physiological brain aging and age-dependent neurodegenerative loss. Future longitudinal studies tracking individual dogs across their lifespan are essential to definitively disentangle age-dependent baseline NfL trajectories from active, pathological neurodegenerative decline.

To establish more evidence-driven diagnosis, we hypothesized that NfL concentrations could be a potential tool for distinguishing CCDS from non-neurodegenerative diseases in the geriatric canine population; however, this hypothesis was rejected in our study. Nevertheless, the high sensitivity of cNfL to morphological brain changes underpins the rationale for developing sNfL as a point-of-care screening tool [40] prior to pursuing more invasive MRI and CSF analysis.

It is worth noting that cNfL and sNfL exhibited similar biological trends in relation to age and BW, although these correlations did not reach statistical significance in the serum cohort. The smaller sample size available for sNfL analysis relative to cNfL inherently widened the confidence intervals and reduced statistical power. Furthermore, compared with the more tightly regulated environment of CSF, NfL concentrations in serum are more susceptible to various physiological factors, as demonstrated in human studies [21,33]. This compartmental susceptibility was particularly evident in our CA group, which displayed markedly greater dispersion in sNfL compared to the relatively tight distribution of cNfL. Under physiological steady-state conditions, NfL concentrations in CSF are approximately 40-fold higher than those in circulating blood [23]. Central NfL enters the peripheral vascular compartment through lymphatic pathways [41]. Once within the vascular system, blood NfL levels represent a dynamic equilibrium governed by CSF influx rates, blood volume dilution (inversely related to body mass), and hepatic and renal clearance [19,22,42,43].

Our SBD cohort exhibited high pathological heterogeneity, encompassing inflammatory disease, cerebrovascular events, aggressive neoplastic lesions, and chronic, slow-growing masses. Since the level of NfL is different between CNS diseases with a different degree of large myelinated axon damage and/or with a different progression rate or diseases intensity [23], this variability likely complicated the interpretation of NfL concentrations and may have contributed to the statistical overlap in cNfL and sNfL levels between the SBD and CA subgroups. Given our sample size, further sub-stratifying SBD into individual neoplastic vs. vascular vs. nerve sheath subgroups would have severely underpowered the statistical analysis. Future investigations focusing specifically on dogs with chronic, slow growing mass lesions and evaluating the differences in NfL levels between these dogs and those with CCDS are warranted, as these two conditions frequently share similar clinical progression and presentation.

It was an unexpected finding that, unlike in a previous study [29], the difference in sNfL concentrations between the SBD and control subgroups was not statistically significant. This lack of statistical significance could be attributed to the bidirectional narrowing of the difference between the two groups. First, the sNfL values in the SBD subgroup may have been skewed downward because the cohort included dogs with chronic, slow-growing neoplastic lesions in a stagnant stage, in which active axonal shedding was less pronounced. Meanwhile, given the age of our control cohort and the fact that validated behavioral questionnaires (e.g., CADES or DISHAA) were not prospective administered across all cases, dogs with unrecorded or mild cognitive impairment that remained undetectable on MRI were likely included in the control subgroup. This may have artificially elevated sNfL concentrations in the control subgroup. To improve diagnostic performance, future prospective studies should combine standardized cognitive questionnaires with blood NfL measurements.

Our study demonstrated that physiological variables should be considered in the clinical application of NfL, particularly in veterinary medicine. Body weight was significantly correlated with cNfL and showed a trend toward statistical significance with sNfL in the >8 y/o group (*p* = 0.053). The substantial variation in body weight across dog breeds may have magnified this inverse association. There was an apparent divergence in breed composition between our study and previous studies evaluating NfL levels in aged dogs [18,19,34]. Most breeds in our study were small- to medium-sized, in contrast to the medium- to large-sized breeds that were more prevalent in previous studies. This difference in breed composition and body size distribution may also have contributed to the higher NfL levels observed in our cases.

Furthermore, we found that the effect of body weight on NfL levels was even stronger in younger dogs, which is consistent with previous findings that the correlation between blood NfL and body weight was only evident in Labrador Retrievers younger than 11 years of age [19]. There is no known mechanism explaining the influence of body weight on cNfL. This was an unexpected finding, considering that small-breed dogs age more slowly than large-breed dogs and that the aging process is associated with increasing NfL levels in body fluids. A similar trend toward an inverse association between cNfL and estimated blood volume calculated from body weight was observed in humans, although the result did not reach statistical significance [33]. We hypothesized that the concentration effect associated with a smaller CSF volume, similar to the effect of blood volume on blood NfL, may be a contributing factor. Another potential explanation is that the chondrodystrophic traits are more prevalent among smaller breeds, and clinically silent intervertebral disk disease may affect NfL levels [19]. The relationship between body weight, cNfL, and blood volume, as well as their combined effects on blood NfL levels, remained to be determined.

In the current study, IRIS stage was not correlated with NfL concentrations, although accumulating evidence in humans indicates that blood NfL levels may be overestimated in the presence of renal impairment. Owing to the retrospective design of our study, IRIS staging was based only on plasma creatinine concentration and did not account for other indicators of renal function. The sarcopenic state of geriatric dogs can underestimate plasma creatinine levels. Further studies using a more comprehensive evaluation of renal function, incorporating symmetric dimethylarginine, urinalysis, and blood pressure measurements, are required to draw definitive conclusions.

Extreme individual outliers were observed in the cNfL cohort. Although neurofilament light chain is structurally stable during ultra-low-temperature storage at −80 °C for up to 11 years in CSF samples [44] and at −20 °C after 12 months in serum samples [45], one report found serum NfL increased after a 6 h delay at 2–8 °C before centrifugation [46]. Pre-analytically, while institutional protocol mandated sample centrifugation within 1 h of acquisition, precise processing time-logs were not maintained for historical biobank specimens, leaving unrecorded pre-freezing handling delays as a potential technical confounder. Clinically, concomitant systemic and axonal pathologies likely contributed to these marked spikes. Notably, one extreme outlier in the CA group represented the sole patient with advanced renal dysfunction (IRIS Stage 3 chronic kidney disease), a condition known to compromise biomarker clearance and elevate serum concentrations in human [35]. Furthermore, two outliers were toy/small-breed dogs (miniature poodle and Shih Tzu), in which unrecorded, concurrent intervertebral disk degeneration may have contributed an additional spinal axonal injury pool to the intrathecal cNfL concentration.

### Limitations

Several limitations of this study should be considered when interpreting our findings. First, although the total biofluid sample size was relatively large (*n* = 126), the subset of geriatric dogs with paired CSF and serum samples was relatively limited (*n* = 25). Additionally, no a priori sample size calculation or statistical power analysis was performed prior to study initiation, as sample recruitment was constrained by the fixed availability of archived historical biobank specimens. Consequently, the limited sample size—particularly within the paired serum analysis cohort—reduced overall statistical power and increased the risk of Type II errors when evaluating subgroup differences. Therefore, sNfL should not be viewed as a standalone diagnostic marker without incorporating age and body weight covariates. Second, the lack of cognitive behavioral evaluations introduces the potential for misclassification within the imaging-based subgroups. The potential presence of unrecorded mild cognitive impairment among older dogs in the control or SBD subgroups cannot be completely ruled out, which may have introduced subclinical variability into baseline biomarker comparisons. CADES scores were not retrospectively assigned due to concerns regarding recall bias. Furthermore, the retrospective design precluded the acquisition of comprehensive physiological variables that could influence NfL concentrations. For instance, height is hypothesized to affect NfL concentrations through its direct influence on total blood volume, an association repeatedly demonstrated in Labrador Retrievers [19,34]. In humans, blood volume can be approximated using body weight alone; however, incorporating height and sex into Nadler’s formula provides a more precise estimate. Whether incorporating height improves blood volume estimation in dogs of similar body weight, or whether height serves as an independent predictor of NfL concentrations, remains to be determined. In clinical veterinary pathology, breed markedly affects reference intervals across complete blood counts, biochemistry, thyroid, and hemostatic panels [47,48]. By extension, circulating NfL concentrations may similarly exhibit distinct, breed-specific baseline reference intervals. Establishing definitive, breed-stratified normative reference intervals across specific purebred lineages will require prospective, multi-institutional studies with larger, age-matched cohorts. Nevertheless, the current study provides a preliminary investigation of NfL application in aging dogs of diverse body sizes. In human clinical neurology, the interpretation of fluid neurofilament levels has already shifted toward the use of age- and disease-specific reference percentiles, supported by online interactive tools to assist clinicians [49]. Future prospective studies should aim for larger sample sizes and more comprehensive collection of physiological factors to improve the diagnostic accuracy of NfL in canine patients.

## 5. Conclusions

Our findings underscore the complexity of using blood NfL as a surrogate for CSF NfL levels. Despite the monotonic correlation observed in the geriatric cohort, raw sNfL concentrations alone demonstrated limited diagnostic utility in discriminating subtle neurodegenerative changes from other structural brain diseases. Notably, as observed in our analysis, the ability of cNfL to discriminate intracranial pathologies from normal controls was enhanced after adjusting for the confounding effects of age and BW. A similar optimization strategy may be applicable to sNfL; identifying and systematically controlling confounding physiological factors will be crucial for improving its diagnostic precision and validating its role as a practical, non-invasive screening tool to justify brain MRI and CSF analysis despite the inherent risks associated with general anesthesia.

## Figures and Tables

**Table 1:** Descriptive statistics and clinical diagnoses of the four imaging-based subgroups in the >8-year-old cohort with cerebrospinal fluid neurofilament light chain measurements.

Subgroup	Structural Brain Disease (*n* = 25)	Cerebral Atrophy (*n* = 18)	Mixed (*n* = 8)	Control (*n* = 18)
Age (years) *	11 (10.5–12.0)	14 (13.5–15.3)	13.5 (13.0–15.0)	11.0 (9.75–13.0)
BW (kg) *	12.5 (6.6–20.4)	7.2 (4.72–12.28)	7.22 (5.95–8.55)	17.40 (8.35–21.73)
IRIS stage (n)	Stage 1 (22) Stage 2 (3)	Stage 1 (13) Stage 2 (4) Stage 3 (1)	Stage 1 (6) Stage 2 (2)	Stage 1 (15) Stage 2 (3)
NfL level (pg/mL) *	3419.65 (2362.83–7418.68)	7949.79 (5337.75–10,766.78)	9554.00 (5780.77–23,250.19)	2406.10 (1516.40–3017.19)
Diagnosis (n)	Glioma (11) Meningioma (8) TNST (2) Infarction (1) MUE (1) LSA (2)	CCDS (17) CCDS/IVS (1)	Meningioma/CCDS (6) Glioma/CCDS (1) Hematoma/CCDS (1)	Cryptogenic epilepsy (7) IVS (6) IFP (4) SARD (1)

* The results are expressed as medians and interquartile ranges. BW, body weight; CCDS, canine cognitive dysfunction syndrome; IFP, idiopathic facial paralysis; IRIS, International Renal Interest Society; IVS, idiopathic vestibular syndrome; LSA, lymphosarcoma; MUE, meningoencephalitis of unknown etiology; NfL, neurofilament light chain; SARD, sudden acquired retinal degeneration; TNST, trigeminal nerve sheath tumor.

**Table 2:** Descriptive statistics and clinical diagnoses of the four imaging-based subgroups in the >8-year-old cohort with serum neurofilament light chain measurements.

Subgroup	Structural Brain Disease (*n* = 9)	Cerebral Atrophy (*n* = 5)	Mixed (*n* = 1)	Control (*n* = 10)
Age (years) *	11.0 (11.0–12.5)	15.0 (13.0–160)	13	10.5 (9.0–12.25)
BW (kg) *	9.15 (5.85–14.8)	7.80 (4.51–15.05)	7.2	17.4 (10.35–20.98)
IRIS stage (n)	Stage 1 (7) Stage 2 (2)	Stage 1 (5)	Stage 2 (1)	Stage 1 (7) Stage 2 (3)
NfL level (pg/mL) *	78.04 (52.25–102.08)	169.63 (101.05–367.92)	648.85	67.98 (21.12–80.72)
Diagnosis (n)	Meningioma (3) LSA (1) TNST (2) Glioma (1) Hematoma (1) Infarction (1)	CCDS (4) CCDS/IVS (1)	Meningioma/CCDS (1)	Cryptogenic epilepsy (4) IVS (3) IFP (2) SARD (1)

* The results are expressed as medians and interquartile ranges. BW, body weight; CCDS, canine cognitive dysfunction syndrome; IFP, idiopathic facial paralysis; IRIS, International Renal Interest Society; IVS, idiopathic vestibular syndrome; LSA, lymphosarcoma; NfL, neurofilament light chain; SARD, sudden acquired retinal degeneration; TNST, trigeminal nerve sheath tumor.

## Data Availability

The original contributions presented in this study are included in the Supplementary Materials. Further inquiries can be directed to the corresponding author.

## Supplementary Materials

The following supporting information can be downloaded at: https://www.mdpi.com/article/10.3390/ani16172659/s1, Table S1. Data file.

## Abbreviations

The following abbreviations are used in this manuscript:

AD	Alzheimer’s disease
BW	Body weight
CA	Cerebral atrophy
CCDS	Canine cognitive dysfunction syndrome
CI	Confidence interval
CKD	Chronic kidney disease
cNfL	Cerebrospinal fluid neurofilament light chain
CNS	Central nervous system
CSF	Cerebrospinal fluid
DISHAA	Disorientation, social interaction, sleep disturbance, house soiling, learning/memory, and anxiety
GLM	Generalized linear model
IFP	Idiopathic facial paralysis
IHA	Interthalamic adhesion
IQR	Interquartile range
IRIS	International Renal Interest Society
IVS	Idiopathic vestibular syndrome
LOESS	Locally weighted smoothing
LSA	Lymphosarcoma
MR/MRI	Magnetic resonance/magnetic resonance imaging
MUE	Meningoencephalitis of unknown etiology
NfL	Neurofilament light chain
SARD	Sudden acquired retinal degeneration
SBD	Structural brain disease
Simoa	Single-molecule array
sNfL	Serum neurofilament light chain
T1W	T1-weighted
T2W	T2-weighted
TNST	Trigeminal nerve sheath tumor
