# Supplementary material for: Investigation of Serum Neurofilament Light Chain as a Surrogate for Cerebrospinal Fluid Neurofilament Light Chain in Canine Cognitive Dysfunction Syndrome
Source: Animals (Basel). 2026 Aug 25;16(17):2659. doi: 10.3390/ani16172659 (PMC13564329; doi:10.3390/ani16172659)
Supplement: Supplementary file 1 [file animals-16-02659-s001.zip › animals-4470586-supplementary.pdf]

Table S1. Data file

Case	cNFL	sNFL	Sub-group*	Age	BW	Breed	Sex#	IRIS stage
1	24722.06		1	9.0	27.0	1	3	1
2	1789.05		1	11.0	15.9	2	3	1
3	2403.30		1	12.0	12.1	2	3	1
4	1213.78		1	8.1	12.9	2	4	1
5	2912.68		1	12.0	5.5	3	3	1
6	2322.36		1	9.0	12.5	2	1	2
7	5914.22		1	12.0	5.2	3	1	1
8	29119.34		1	14.0	9.5	4	1	1
9	1560.46		1	9.0	14.0	2	3	1
10	21675.33		1	8.2	11.0	5	3	1
11	3671.84		1	11.0	33.0	6	1	1
12	8489.15		1	12.0	29.1	6	3	1
13	3209.33		1	11.0	36.0	6	4	1
14	24298.82		1	19.8	20.7	1	4	1
15	6348.20		1	13.0	20.1	7	3	1
16	1202.70	43.97	1	11.0	9.2	18	1	1
17	2403.53	64.03	1	14.0	16.1	1	3	2
18	3419.65	78.04	1	12.0	6.0	3	3	1
19	4421.03	110.19	1	11.0	10.9	3	4	1
20	4929.09	48.49	1	12.0	13.5	9	1	1
21	3263.79	93.96	1	10.0	5.7	3	3	1
22	5931.15	83.63	1	11.0	7.2	3	3	1
23	14669.55	312.65	1	11.0	4.4	2	1	1
24	2086.54	56.01	1	13.0	24.4	1	3	2
25	2680.76		1	12.0	5.1	3	3	1
26	11450.29		2	15.0	6.2	8	3	2
27	7292.56		2	14.0	8.4	8	3	1
28	3459.67		2	14.0	13.1	1	1	2
29	14445.99		2	14.0	10.6	1	4	1
30	21347.20		2	15.0	12.0	9	1	3
31	8510.89		2	14.0	2.6	3	1	2
32	5062.24		2	16.0	8.6	11	3	2
33	120353.27		2	14.0	5.5	3	3	1
34	7958.25		2	11.0	20.6	1	3	1
35	9904.50		2	15.0	4.6	3	3	1
36	4064.78		2	9.0	4.8	10	4	1
37	7935.32		2	12.0	6.6	8	1	1
38	3890.44		2	16.0	3.5	3	4	1
39	6413.39	64.51	2	16.0	16.1	9	1	1
40	9034.98	509.84	2	12.0	2.8	10	3	1
41	5429.58	225.99	2	14.0	7.8	9	3	1
42	6624.41	169.63	2	15.0	6.3	9	1	1
43	6685.41		3	15.0	7.8	5	4	1
44	25838.05		3	13.0	5.6	3	1	1
45	5894.68		3	16.0	3.2	3	4	1
46	180472.57		3	14.0	8.8	16	4	1
47	5742.80		3	15.0	15.5	1	3	1

48	5160.61		3	13.0	7.0	3	3	1
49	10538.94	137.58	2	16.0	14.0	4	1	1
50	15486.62	648.85	3	13.0	7.2	8	3	2
51	12422.58		3	13.0	7.2	8	3	2
52	6956.50		4	13.0	6.4	11	4	1
53	4864.90		4	14.0	3.8	3	1	1
54	3252.13		4	11.0	28.8	6	1	1
55	2455.47		4	10.0	9.0	2	2	1
56	1189.31		4	10.0	6.4	11	3	1
57	1828.81		4	12.0	31.5	1	3	1
58	2588.39		4	13.0	20.0	1	4	1
59	2356.73		4	11.0	28.5	12	1	1
60	1520.21	76.87	4	12.0	10.2	9	1	2
61	2936.91	74.47	4	13.0	17.6	1	1	1
62	1175.61	200.44	4	10.0	22.4	1	1	2
63	764.93	11.51	4	9.0	4.9	19	3	1
64	3770.49	61.52	4	12.0	21.5	1	2	1
65	1504.97	21.23	4	9.0	20.8	7	3	1
66	2544.56	74.44	4	13.0	10.4	15	1	1
67	1872.92	92.27	4	9.0	20.7	7	1	1
68	2938.87	20.79	4	9.0	15.6	9	3	2
69	1544.22	44.20	4	11.0	17.2	1	3	1
70	330.81	7.89	4	6.0	10.4	9	3	1
71	1004.37	6.11	4	3.0	20.3	1	3	1
72	83368.26	418.38	1	3.0	6.3	5	3	1
73	561.61	8.48	4	3.0	26.1	12	3	1
74	1494.52	5.43	4	3.0	30.2	13	3	1
75	2020.48	28.67	4	6.0	5.9	15	1	1
76	690.14	141.19	4	6.0	13.2	4	3	1
77	5108.69	21.33	1	6.0	10.1	2	3	1
78	831.45	1.52	1	1.0	49.6	17	2	1
79	941.64	8.23	4	4.0	19.9	7	3	1
80	462.51	7.03	4	7.0	12.7	4	3	1
81	5052.53	17.18	4	7.0	6.0	15	1	1
82	4067.54	66.48	1	7.0	8.9	20	1	1
83	1109.08	28.49	4	6.0	6.3	15	1	1
84	10890.90	92.12	4	6.0	2.7	14	4	1
85	649.65	2.16	4	.7	14.4	7	2	1

* 1: Structural brain disease; 2: Cerebral atrophy; 3: Mixed; 4: Control

1: Spayed female; 2: intact female; 3: neutered male; 4: intact male
